# Governing COVID-19: analyzing the effects of policy responses on food systems in Tanzania

**DOI:** 10.1186/s40066-022-00383-4

**Published:** 2022-09-09

**Authors:** Paschal A. Mugabe, Theresa M. Renkamp, Constance Rybak, Hadija Mbwana, Chris Gordon, Stefan Sieber, Katharina Löhr

**Affiliations:** 1 University of Dar Es Salaam, P.O. Box 35091, Dar es Salaam, Tanzania; 2 Leibniz Centre for Agricultural Landscape Research, Eberswalder 84, 15374 Muncheberg, Germany; 3 Sokoine University, Chuo Kikuu, P.O. Box 3000, Morogoro, Tanzania; 4 University of Ghana, P.O. Box 29 LG, Accra, Ghana; 5 Humboldt-Universität zu Berlin, Invalidenstr. 42, 10115 Berlin, Germany

**Keywords:** COVID-19, Smallholder farmers, Food systems, Policy responses

## Abstract

**Background:**

The outbreak of the COVID-19 pandemic increased debates on global public health concerns. From early 2020 to 2022, globally, life was upended in the wake of the pandemic. Industries of all kinds were forced to rapidly changed how they work, including agriculture. Particularly for smallholder farmers in developing countries, the COVID-19 pandemic, coupled with climate change effects, negatively affected their livelihoods. Achieving the UN Sustainable Development Goals by 2030 is unrealistic if immediate efforts are not made to address the existential threats facing smallholder farmers. This study analyzes COVID-19 governance and policy responses, examining its effects on smallholder farmers in the south and east of Tanzania using both qualitative and quantitative techniques.

**Results:**

Findings show that mobility restrictions imposed by other countries and fears of the Tanzanian people leading to voluntary isolation resulted in an amended structure of farmers’ markets: Reductions in exports, imports and in the purchasing power of the local people followed. Food security was diminished as food availability on the market level was reduced due to mobility restrictions. The impact of COVID-19 resulted in more than 85% of smallholder farmers experiencing an income reduction, thus also increasing the pre-existing vulnerability of these communities. Findings show that farms producing non-exported crops had less severe income reductions and could cope better. The results indicate that only 20% of smallholder farmers started using digital information technology to gather information since physical movements were restricted. Access to technology remained limited in rural areas. Even during the COVID-19 crises, farmers’ concerns about the vulnerability of their food systems include non-COVID-19 causes, such as climate change.

**Conclusions:**

Although Tanzania did not impose a total lockdown, the country was affected by COVID-19, partly via policies of other countries. Impacts included: (i) a decline in local markets as smallholder farmers had fewer trading partners from neighboring states; (ii) a loss of employment opportunities due to the absence of both local and external trade; (iii) reductions of farm output and income; (iv) a lack of agricultural inputs (fertilizer etc.) that are usually imported; (v) fear to continue farming activities due to news about COVID-19 spreading; and (vi) reduction of work efficiency because of a lack of social gathering due to voluntary isolation.

While COVID-19 compelled policymakers to make urgent decisions to ensure stable food supply chains, the fundamental task is to address these immediate disruptions while also investing in the long-term goal of a resilient, sustainable, and productive global food system. This can be achieved by adopting a policy package that includes investments in technological development, access to small long-term loans, and regulatory reforms, with which governments can create conditions supporting productive, sustainable, and resilient food systems that can withstand future shocks.

## Introduction

Beyond the public-health crisis triggered by the COVID-19 pandemic, restrictions on the economy and society are drastically affecting human development by pushing people into poverty, increasing mental-health complaints, and curtailing children’s access to both education and healthcare [24]. Nearly, 2 years in, the COVID-19 pandemic remains front-and-center in global conversations. From vaccine rollout challenges and the rise of multiple variants to indefinitely disrupted day-to-day operations across sectors, the virus continues to loom large. The spread of COVID-19 has deepened the vulnerability of millions of small producers and agricultural workers, leading to intensified uncertainty for a large part of the world’s population [8].

While global leaders seek to vaccinate and recover economically, there is also a shared urgency to build momentum around the Sustainable Development Goals. The crisis is a global wake-up call for the need to redouble efforts to work together to solve shared challenges. Every disease affects not only people’s health but comes with direct and indirect effects on socioeconomic status, agriculture, food security, and dietary intake [18]. Agriculture is one of the most essential sectors for human development and is directly related to food security [1, 10, 12]. The rapidly spreading COVID-19 outbreak has tested the resilience of food supply chains even in the European Union (EU) [27].

On January 30, 2020, the WHO declared COVID-19 a public health emergency of international concern, calling for the world to take it seriously. Between December 2019, when the first case of the disease was reported in China, and the end of December 2021, more than 274 million confirmed infections and 5 million deaths were recorded [25]. The Global COVID-19 Humanitarian Response Plan was revised significantly upwards to reflect the increasingly urgent need to address the non-health impacts of COVID-19. Of these needs, food security represents the largest component, requiring a total of USD 1.6 billion [7].

Globally, the COVID-19 pandemic has already directly affected food systems, affecting food supply and demand, as well as indirectly through reduced purchasing power, reduced capacity to produce and distribute food, as well as the intensification of care tasks, each having differentiated impacts and all more strongly affecting the poor and vulnerable [7]. Emerging information on the interactions between the COVID-19 pandemic and global food systems highlights how the pandemic is exacerbating food crises across Africa [23]. Less clear, however, are how the impacts differ between farming systems and how different governments take different policy measures in relation to the pandemic.

In many respects, populations and food systems across Africa are less vulnerable to COVID-19, compared to other regions in the world [16], given the persistence of subsistence food production, shorter food supply chains, lower rates of urbanization, and lower population densities in many areas, alongside a youthful population [13]. However, key vulnerabilities stem from high and chronic levels of poverty [6], a reliance on food import–export markets, the informal nature of local food supply chains, as well as impracticalities with social distancing and lockdowns among labor-intensive farming systems and informal economies [11].

For Tanzania, food security and dietary intake have not been exempted from the disruptions of the outbreak of COVID-19. Understanding the implications of COVID-19 and associated restrictions is necessary to foster a sustainable endemic recovery for farmers and farm systems. Investigating the governance role, how COVID-19 impacted local-scale food production, and how farmers adapted, can support a better understanding of farmers’ resilience. In turn, this can help inform coherent responses for a sustainable post-COVID-19 recovery. In response to calls for more resilient farming and food systems, our study aims to explore the implications of policy responses to COVID-19 as well as associated national restrictions on-farm systems for smallholder farmers in the Southern Highlands of Tanzania.

### Motivation of the Tanzania case study and justification of the study

Tanzania, once known as an anti-coronavirus nation, drastically changed its stance on the virus following the death of President John Magufuli, an ardent coronavirus skeptic who touted prayer as the best remedy. The East African country, which largely eschewed mask-wearing and social distancing, joined the World Health Organization’s COVAX initiative through which it received more than 1 million doses of the Johnson & Johnson (Janssen) vaccine donated by the US. Tanzania did not undergo any lock-down measure against COVID-19 as of December 2021, apart from closing schools for 2 months. Residents, both rural and urban, continued with their daily routine as normal throughout the first 2 years of the pandemic.

However, Tanzania did not escape from the impact of the pandemic despite its stance on the pandemic. With a shift in policies due to the new government, Tanzania, in particular, presents an interesting case. Furthermore, looking at the structure of food production in Tanzania, 90 percent of food supplies are produced by the rural poor [2]. This raises the question of how are the rural poor affected by COVID-19? This study illuminates the links between governance in curbing COVID-19 and the impact on smallholder farmers. It also explores sustainable ways to strengthen the resilience of the most vulnerable communities.

### Background information about COVID-19 and its regulations in Tanzania

The confirmed number of people who tested positive for COVID-19 in Tanzania until May 8, 2020, was 509, including 21 who died from the disease. Thereafter, no cases were reported for approximately 1 year. Nine regions were considered to be high-risk due to their proximity to border points of entry, connection to international flights, and the location of the initial COVID-19 cases [21]. Following the country’s first reported case, a 30 day ban was imposed on public gatherings (except for worship) and the schools were closed. On April 17, 2020, the Government extended the school closure indefinitely. Zanzibar banned all tourist flights from entering the region and authorities in Kigoma Region advised refugees to stay inside the camps.

Tanzania then suspended all international commercial flights on April 12, 2020. However, on May 14, some flight restrictions were lifted for repatriations, humanitarian aid, medical cargo, and relief flights, as well as other safety-related operations. Then, on May 18, the passenger flight suspension on flights to and from Tanzania was lifted completely. Tanzania maintained open land borders throughout the COVID-19 outbreak. On June 8, 2020, the Government of Tanzania officially declared the country to be free of COVID-19 and all restrictions were lifted. Nevertheless, neighboring countries were feeling the threat of the pandemic, all of which are key trading partners. Their continuing COVID-19 control measures disrupted regional and domestic agricultural markets, affecting local livelihoods and food systems.

After the death of President Dr. Magufuli, Tanzania adopted a new approach toward fighting COVID-19. With the formation of the new cabinet on April 6, 2021, President Samia Suluhu Hassan announced her plan to form a technical committee to give recommendations on how the country could curb the spread of the COVID-19 pandemic. On May 17, 2021, the appointed committee came up with resolutions that included requesting the government to develop a contingency and response plan for COVID-19, make official announcements about the presence of the pandemic, provide clear statistics about the spread of the pandemic, and direct the public to take all precautionary measures.

Accordingly, Tanzania should effectively be involved in regional and international resolutions agreed upon through the East African Community (EAC), Southern African Development Cooperation (SADC), African Union, and World Health Organization (WHO). Furthermore, the resolution states that the public is free to choose whether to take vaccination or not. Since then, Tanzania joined the global community in fighting against the COVID-19 pandemic and is adhering to all international standards and recommendations.

Since June 2021, cases have been reported, but the report of numbers appear sporadic in most months with the exception of a strong increase in October 2021. As of December 2021, there were 26,483 cumulative cases, of which 25,569 were still reported to be active. This hints at the fact that the case numbers were not updated as recovered cases are neither identified nor counted. Overall, 734 deaths were reported as of December 2021 [26].

## Methodology

### Research approach

By employing an exploratory design, this study creates knowledge that further investigations can build upon. For the study, a two-step mixed methods approach was chosen, combining quantitative and qualitative research tools. This approach was chosen with the aim to (i) explore more broadly the impacts of COVID-19 on smallholder farmers in Tanzania using quantitative web-based survey questionnaires before (ii) gaining more in-depth understanding through qualitative individual semi-structured interviews. The first part consists of a quantitative analysis based on a questionnaire that was distributed in December 2020.

The questionnaires were structured and contained mostly closed questions about the impact of the outbreak of COVID-19 on food security, agricultural activities, community support, governmental support, coping strategies, and the use of Information Technology (IT). The survey was distributed online as well as face-to-face. The selection of interview partners was purposive. Not only were experts from agricultural and government organizations involved, but also village leaders and smallholder farmers, taking into account age and gender balance during the selection.

To deepen knowledge of the pandemic’s impacts on Tanzanian smallholders, personal semi-structured interviews were conducted with smallholder farmers. Interview questions resembled a descriptive design that generated a profile of described relevant aspects of the phenomena of interest from individual, organizational, and industry-oriented perspectives. Thus, it enabled the team to gather data from a range of respondents. In addition, a literature review is employed as a source of secondary data, reviewing articles, reports, books, and official documents of the Tanzanian government related to COVID-19.

All interviewees were informed about data protection issues by the enumerators and gave their consent orally at the beginning of each interview. Data was analyzed and presented in anonymous and aggregated form.

### Selection of study areas

**Fig. 1:**
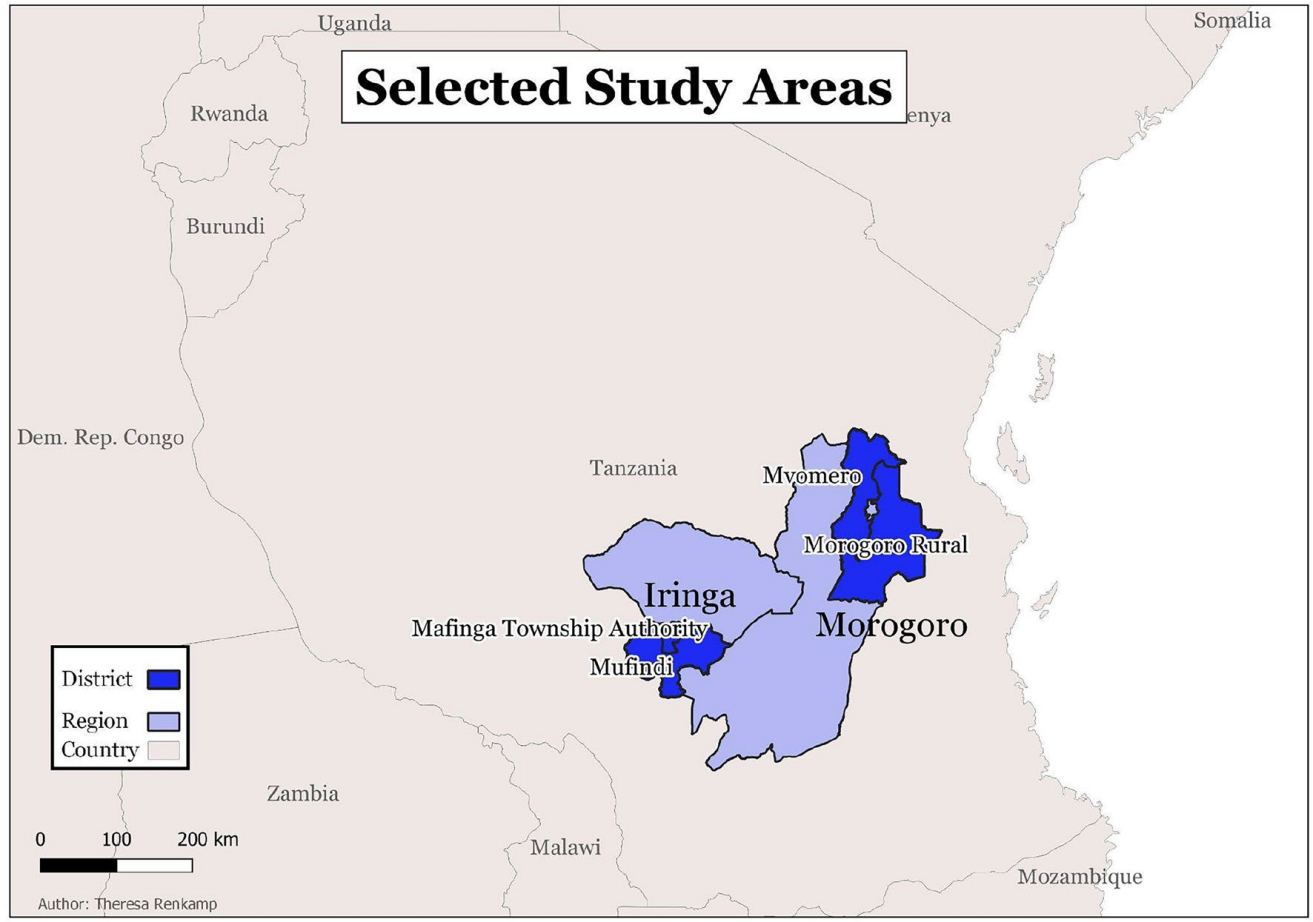
Selected study areas in Tanzania

The target area for the quantitative data collections was Morogoro Rural and Mvomero, districts of Morogoro region in eastern Tanzania. Three villages in close vicinity to Morogoro, namely, Kiroka in Morogoro Rural District as well as Tangeni and Mvomero were selected as study sites (as indicated in Fig. 1). These sites were selected, because they represent typical Tanzania rural smallholder farming systems and have a strategic position in terms of trade as Morogoro is an inlet and outlet of trade between Dar es Salaam and other regions, such as Dodoma, Iringa, Njombe, and Mbeya, as well as between neighboring countries, such as Zambia and Malawi. The smallholder farmers cultivate 90% of the land in the region and contribute 75% of total agricultural outputs [22].

The second selected municipality was Mafinga in Mufindi District, Iringa, Southern Highlands of Tanzania. Changarawe village (Sao Hill Ward, Ifwagi Division) and Kikombo village (Isalavanu ward, Sadani Division) were selected as study sites (Fig. 1) as they represent a typical Tanzanian rural economy with smallholder agricultural activity predominating. With most land covered by forest, Mafinga Municipal is also a commercial hub that is known for trading timber and tea internationally. Thus, it provides a good example of how travel restrictions have affected these communities in terms of selling their products during the COVID-19 pandemic.

### Demographic structure of the respondents

The overall sample size is 59. Regarding the sample in Changarawe and Kikombo villages (Mafinga, Iringa), purposive sampling was used to select a sample of 20 village respondents (ii). The purposive sampling technique was employed in order to obtain a balanced sample of different types of smallholder farmers in the two villages. For Morogoro, the web-based questionnaire (i) was conducted, 77% of respondents are smallholder farmers (*n* = 30) and 23% are agricultural experts either working in organizations or research institutions (*n* = 9). In this case as well, purposive sampling was used in order to include a balance in gender, education, age, and type of farming, as well as a variety of expert opinions. Out of the interviewed farmers, 50% own less than 2 ha of land and 40% own between 2 and 5 ha. Over 60% of the farmers live more than 10 km away from the city. Furthermore, around 75% of farmers are between 30 and 49 years; 25% are over 50 years. The average household size of the interviewed smallholders is five persons, of which almost 80% have one or two children at school as indicated in Table 1 below.

**Table 1:** Demographic structure of the respondents Morogoro and Mafinga (*n* = 39)

S/N	Villages	Gender		Education		Age		Type of farming	
		M	F	7	14	30–50	50–70	Commercial	Subsistence
1	Changarawe	5	5	4	6	8	2	8	2
2	Kikombo	5	5	7	3	7	3	7	3
3	Morogoro	13	15	24	5	14	15	15	14
	Total	23	25	25	14	29	5	30	19

7 primary education (7 years)

14 secondary education and above (14 years)

## Results & Discussion

### Effects of COVID-19 on-farm production and income

The data show that 45% of farmers experienced reduced farm output due to COVID-19. This output reduction was severe, as 60% of farmers with a reduced output report a decrease of 25%; 40% of farmers experienced a decrease of 50%. COVID-19 had a greater impact on the incomes of smallholder farmers. The quantitative analysis shows (i) that more than three-quarters of respondents faced an income reduction; of which, 35% of farmers reported a 75% decrease and more than 20% saw their income cut in half. From the qualitative data, (ii) about 95% confirm that the pandemic decreased their income due to reduced production and market disruptions. It is notable that the income reduction due to COVID-19 is more severe than the reduction of output. Not only did more smallholders report an income reduction overall, the reductions reported are also greater. Furthermore, a strong negative correlation (r = − 0.43) is found between household size and income decrease, meaning that with larger households, less income loss was reported.

Looking at different types of farming, the data shows that the strongest negative impact of COVID-19 on farm output and farm income (reduction of sales) is in subsistence farming, with 39% of subsistence farmers reporting output reductions of up to 50%, while 54% of all subsistence farmers reported income decreases of 50–75%. Commercial plantation farms showed a significant decrease in farm income, with 63% of commercial farms reporting an income loss of 75%, and 38% reporting an output decrease. One-third of mixed farms reported an income loss of 25%, while 22% reported an income reduction of 50%. Compared to the amount of lost income in commercial and subsistence farming, mixed farms showed the lowest decrease in output and income. Smallholders engaged in mixed farming are, relatively speaking, the least impacted. Mixed farms as well as larger household sizes experienced less income reduction. This can be explained by diversified income sources within larger families but also better social connectivity of larger households leading to a range of advantages and greater overall resilience, as Cassidy and Barnes (2012) show [4].

### Changes in agricultural activities experienced since the outbreak of COVID-19

With regards to quantitative data (i) half (50%) of respondents reported increased difficulties in selling products due to the absence of international trade, the decline of purchasing power and voluntary isolation. Parallel to this, 40% claim that mobile traders can no longer collect produce from farms. Over 40% of respondents claim that the agricultural employment sector has changed. Farmers report that they had to lay off their casual farm laborers go as they could no longer afford to pay them due to the decline of revenue.

The majority of respondents (70%) in Mufindi District (ii) reported that COVID-19 affected farm production because of a loss of farm labor. One respondent in Kikombo village argues that, *“farm hired labor became scarce while hiring labor became difficult for farms,”* as people were afraid of the pandemic after it was announced. Before the outbreak of COVID-19, there was enough farm labor supply. However, during COVID-19, labor became scarce as rural hired laborers were not available due to restricted mobility. Labor scarcity led to expensive labor supply. Another reason why COVID-19 complicated farming activities was that people could no longer be as active and productive as they were previously due to isolation. Even though there was no lockdown, information about the presence of the pandemic implied that people adopted voluntary isolation due to fear. As commented by one respondent at Changarawe village at Mafinga,

*‘’You know our culture; we are used to gathering together, be it on farms or during ceremonies etc.… Now Corona divided us as we cannot gather on the farms and work together, so each and every one of us is working in isolation. This, to a large extent, reduces our effectiveness because we are not used to working in isolation’’.*

Furthermore, qualitative data (ii) uncovers that, in particular, respondents who were engaged in both food and cash crop production (80%) confirmed experiencing changes, such as a reduction of farm inputs and income as well as reduced availability of markets after the outbreak of the pandemic. The majority of respondents mentioned difficulties in terms of market availability as the first reason for the change in farming activities. As the study sites are business hubs of the Tanzania Southern Highlands, respondents engaged in cash crop production explained that the decline of the external market due to border closures was a reason why they had to rely more on local markets. The export of agricultural products, such as maize, tea, sunflower, pyrethrum, tobacco tea and paprika, was impeded. Other respondents explain that even local markets had declined, because people who were employed by external businesses were laid off. One farmer, engaged in horticulture, complained that his perishable products—including watermelon, pineapple, and pawpaw—were rotting, because they were not selling quickly. Moreover, the possibility to sell products was reduced, because business people from neighboring countries were not visiting.

Another change to agricultural practices was the change in focus, from both subsistence and cash crops to solely on subsistence crops. This came about as income fell, with increasingly expensive agricultural inputs and market failure. This was mentioned by smallholders farmers engaged in mixed farming without quantifying how much agricultural inputs prices had increased. However, a few individuals (11%) coming from subsistence farming argued that there were no effects of COVID-19 in terms of farming practices since its outbreak. The reason given was that they always produce little just for subsistence; therefore, they did not see any lockdown effects. A major finding was that 19% of the respondents associated the change in farming patterns with climate change rather than the outbreak of COVID-19. This finding matches with observations in similar studies across different geographies [5, 9, 14].

### Input costs change after the outbreak of COVID-19

**Fig. 2:**
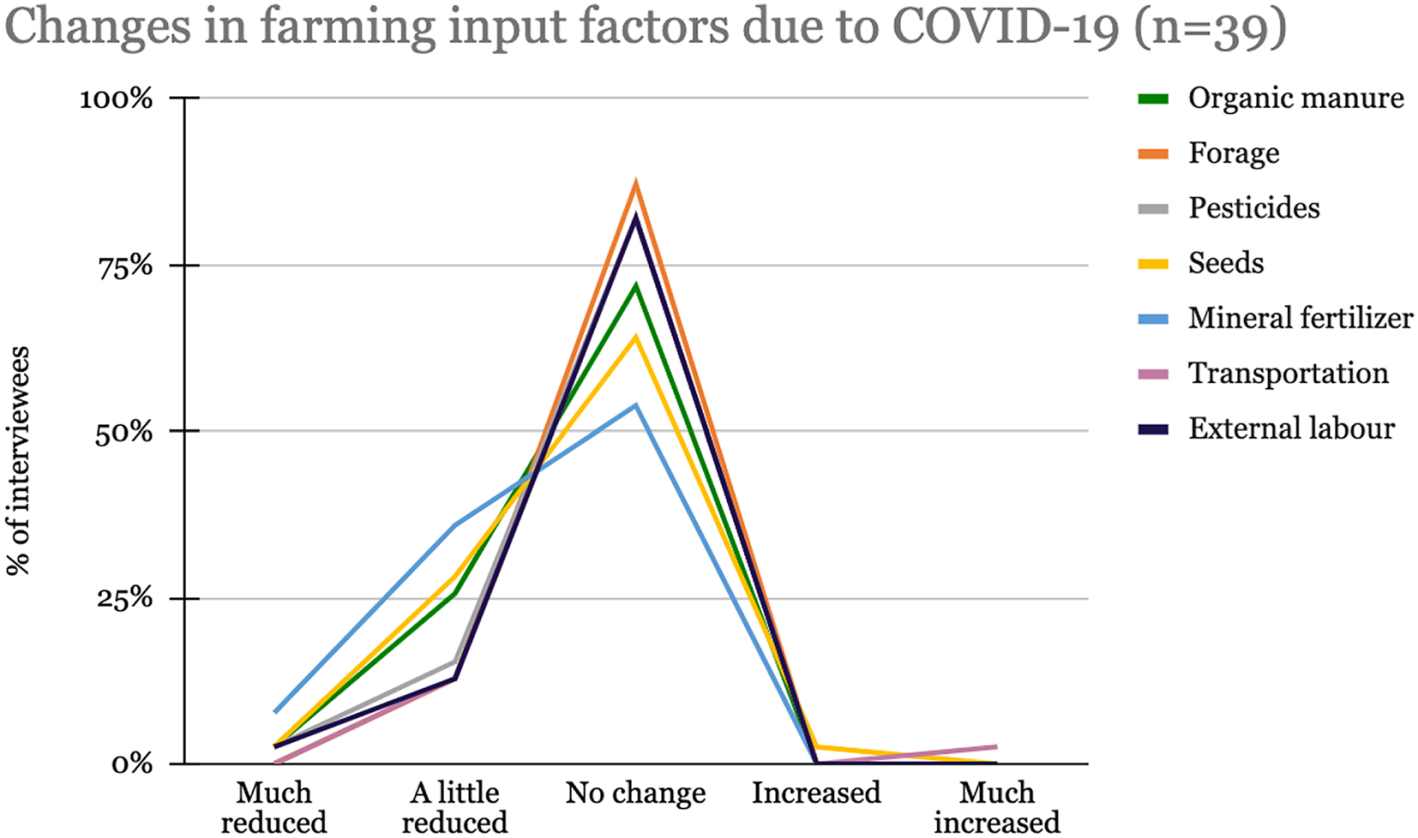
Changes in farming input factors due to COVID-19

As quantitative data below shows (Fig. 2), most respondents did not notice a change in input factors for farming activities. Only for mineral fertilizer, seeds, and organic manure is a (small) reduction described.

Interestingly, qualitative data analysis reveals that all interviewed community members report a scarcity of, and price increase in, agricultural inputs during the pandemic. A reason given was that most agricultural inputs in Tanzania are imported, such that the border closures affected the availability and, therefore, the price of agricultural inputs for farmers in Tanzania.

Thus, while respondents from Morogoro saw almost no change in input factors, Mafinga farmers reported a price increase that resulted in shortages affecting their farming activities. These findings can be explained by the fact that some rural interviewees were small business individuals who could not directly feel the impact of change in input factors. In addition, for the case of Morogoro, about 23% of interviewed (experts) did not see any change in input prices. Although our findings reveal that the prices for agricultural inputs did not increase much, smallholder farmers still faced challenges due to decreased income, reduced output, and the limited provision of both water and fertilizers. Emerging evidence shows that COVID-19 is affecting agricultural activities in Sub-Saharan Africa by disrupting supply chains, labor mobility, labor availability, as well as access to essential farm inputs, such as fertilizers and pesticides [3]. The research shows how COVID-19 and its governing affected food systems in Tanzania, similar to the results of Tripathi et al. (2021) [23], which investigated the impacts of COVID-19 on diverse farm systems in Tanzania and South Africa [23]. Their findings highlight how COVID-19 restrictions interrupted the supply chains of agricultural inputs and commodities, increasing the storage time for produce, decreasing income and purchasing power, while also reducing labor availability. Having found similar results with regards to storage problems, decreased income, reduced purchasing power, and limited labor availability, this study provides further details about smallholder farmers: Next to an income decrease, smallholder farmers also experienced a noticeable output reduction.

### Change in food consumption due to COVID-19

Overall, smallholder farmers experienced changes in food consumption and food security. The strongest reduction was food availability, with availability reduced or much reduced for 58% of the respondents. The qualitative data (ii) shows that more than 78% of respondents did not notice a change in the way they prepared (balanced) meals as they still needed three meals per day as usual. Here, 33% of respondents reported that reduced food availability occurred because of income reduction, while 17% of smallholders reported having difficulties in going to food markets due to mobility restrictions imposed by the government. Another 18% reported that the pandemic changed their food patterns, arguing that they were cooking what remained from their harvest, whereas prior to the pandemic outbreak, they could rotate with marketed food.

Like the study of Nchanji et al. (2021) [17] on bean farmers in central, eastern, and southern Africa, this study investigates the implications of the aforementioned changes on food security for smallholder farmers in Tanzania, asking for policy recommendations to help alleviate poverty and build resilience. Similar to Nchanji et al. (2021) [17], production and distribution challenges affected food consumption, mostly via reduced food availability and increased poverty, with 85% of smallholder farmers seeing their income reduced and food availability being reduced or much reduced for 58% of the respondents.

A (strong) increase in food prices was noted by 40% of respondents; however, another 40% did not see a change in prices and around 20% saw reduced prices. About 15% of respondents from Morogoro reported that the prices for products like oil and fats, sugar, meat, bread and rice had increased. Qualitative data (ii) also reveals that there was a decrease in food prices. Looking at the annual change in wholesale prices for selected food crops, Fig. 4 indicates a decrease in food prices during the pandemic among selected crops: maize, beans, rice, sorghum, round potatoes, and finger millet. In contrast to some respondents’ perception in Morogoro, the price of rice actually decreased during the first year of the pandemic.

**Fig. 3:**
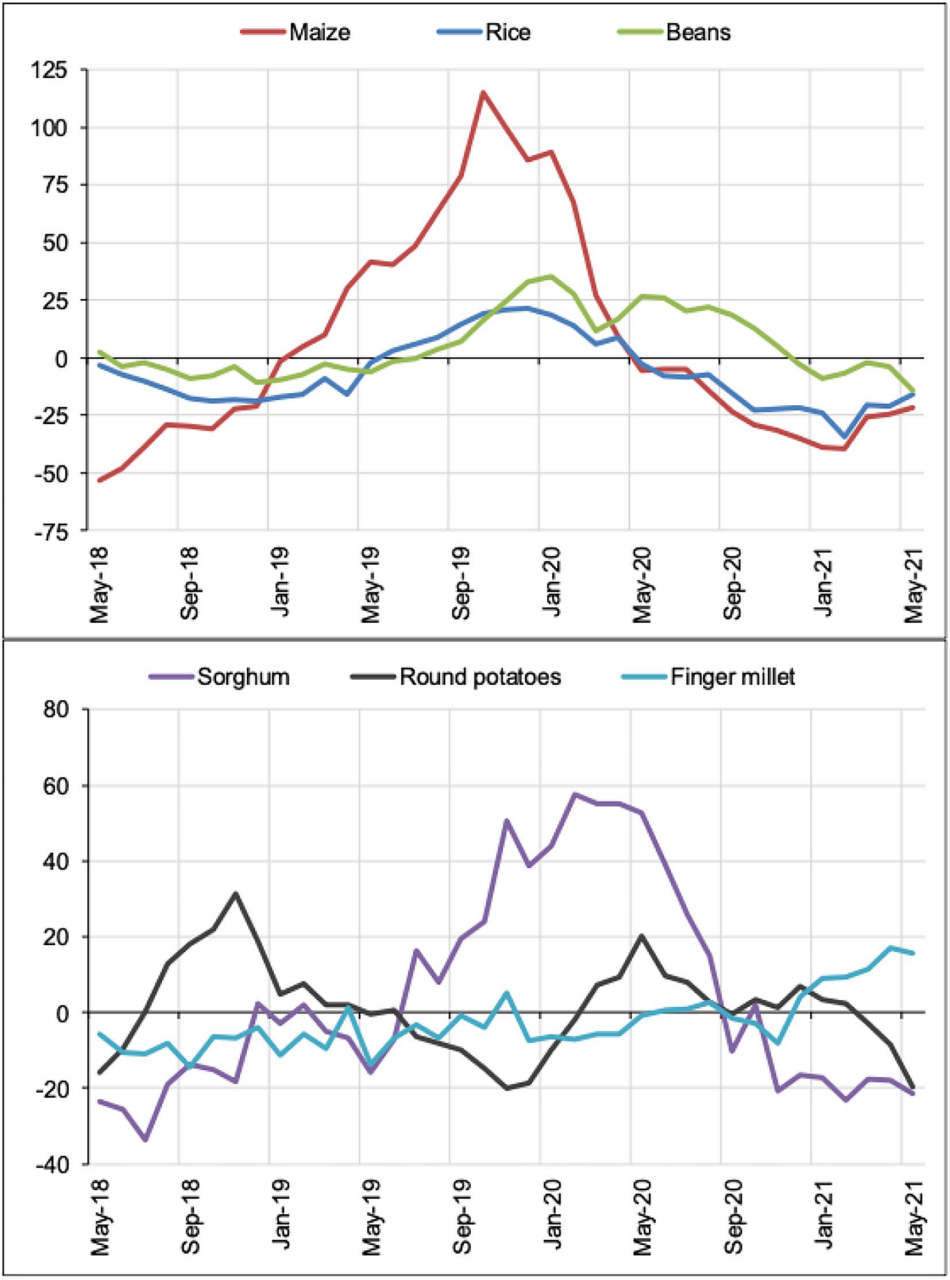
Annual change in wholesale prices for selected food crops [15]

The analysis reveals different perceptions of changes in food prices: While 40% of smallholder farmers in Morogoro reported increased food prices, in particular for oil, fats, sugar, and meat, qualitative data from Mafinga and official data show how there was a strong decrease in prices for most food crops (Fig. 3). On the one hand, products that are imported can be more expensive due to border closures. On the other hand, the perceived price increases by some smallholder farmers might be the result of their lost purchasing power, which made the share of expenses for food relatively greater.

### Changes related to social relationships, interactions and networking

Qualitative data (ii) shows that most respondents noticed a change in social interactions overall. Some respondents claimed that between March and May 2020 people did not gather; rather later, after the government declared that Tanzania was free from COVID-19, the situation changed and people returned to their normal routines. More than half of the respondents confirmed that there was a difference in terms of community gathering between the period before, during, and after the declaration. Gathering patterns play an important role in most rural production systems in Tanzania. In order to speed up farm preparations, households hold official ceremonies by inviting neighbors who spend the whole day assisting to prepare the farms. Instead of depending on the limited labor force of one household, this routine goes from family to family, saving time and preparing all farms for cropping. COVID-19 affected this pattern of community cooperation.

However, according to one respondent in Kikombo Village, social relationships and networks resulting from the outbreak of COVID-19 did not bring any changes as community members were still gathering and it was difficult to keep people at distance. He added that there was not a single COVID-19 case in his community, making the virus an abstract thing, which he only heard about in relation to other countries on the news.

Regarding the extent of interactions among smallholder farmers since the outbreak of the COVID-19 pandemic, more than half of smallholders confirm that a significant change was noticeable. They added that farm activities were eventually stopped as people remained in their houses to avoid any sort of interaction. At the same time, about 35% of respondents argued that they could not witness any change in interactions among farmers as they inform that it is normal for everyone to be in his/her farm.

### Government's support for the agricultural sector

Both quantitative and qualitative data reveal that smallholder farmers did not receive any support from the government of Tanzania. Smallholder farmers explained that their farms faced pests including beetles, bugs, aphids, flies, moths, butterflies, and nematodes that affected their plants and would have liked help from the government. They also complained about high prices of agricultural inputs, such as fertilizers and increased water supply, as challenges for which they did not receive any governmental assistance.

Furthermore, smallholders complained about their dependency on certain middlemen, who, they claimed, could not be avoided because of a lack of choice. Middlemen are condemned for purchasing small farmers' produce at very low prices and selling them at higher prices. Lastly, they also complained about the danger of foreigners who come to grab land and establish tree plantations, which they do not see any benefit from.

### Positive changes experienced since the pandemic

The study also sought to determine if any positive changes at the individual, community, or agricultural level resulted from the outbreak of the COVID-19 pandemic. The vast majority of respondents (90%) said that COVID-19 did not bring any positive change. On the contrary, negative changes, including fear, insecurity, market declines, social isolation, and general hardships are reported. Only 10% of the respondents mention that they saw some positive changes as a result of the pandemic. Among the positive changes mentioned was the decrease in food prices, because people did not have purchasing power during the pandemic. Some respondents mentioned learning to use digital technology for information gathering, since physical movements were restricted. Another two respondents said that they did not see any positive developments, since the government was not giving any support to the farmers and climate change was a major problem of agricultural production.

### Coping mechanisms

**Fig. 4:**
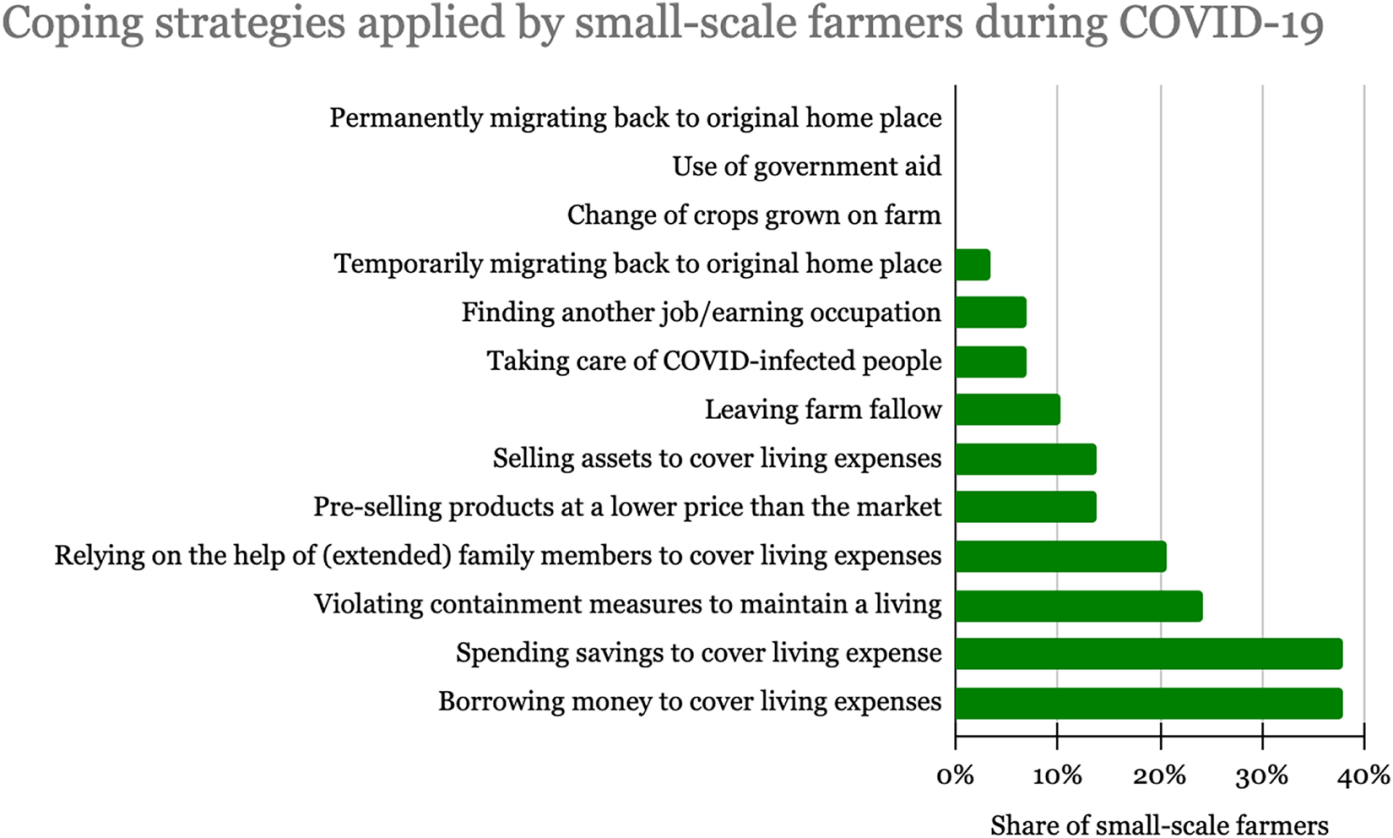
Coping strategies applied by farmers during COVID-19. Source, survey data 2021

Smallholders mostly indicate using financial strategies to cope with the crisis: 38% borrowed money or used their savings to cover living expenses. Another 23% of farmers reported that they had to violate the containment measures to maintain their living, while 21% relied on financial help from family members. Next to these financial coping strategies, 15% of smallholder farmers changed production from cash crops to subsistence farming. Others explain that they changed their farming by reducing the size of cultivated farmland to avoid costs. One farmer reported that he stopped producing perishable goods, because there was no market for them. Figure 4 indicates strategies of community members in Morogoro to cope with the new situation.

The qualitative data shows that respondents had divided opinions about this question. A large part of the respondents (60%) associate their decisions to cope with the situation with a spiritual orientation, stating that they thanked God for being the only thing they could rely on during the crisis. Others (35%) explain that they adapt to the new situation by following government directives to keep distance, wear masks, and regularly wash hands.

Like Tripathi et al. (2021) [23], this study also analyses the responses of farmers to the pandemic situation. Both studies show that farmers’ responses were heterogeneous. Tripathi et al. (2021) [23] show how smallholders shared labor, sold assets, and diversified their farming activities to subsistence produce in order to cope. Similar to our results, cash crop farms focused more on subsistence farming in order to cope with income losses. However, the results of Tripathi et al. (2021) [23], showing those less engaged with international markets being least affected by the associated COVID-19 measures, cannot be fully confirmed for smallholders in both Tanzanian regions, as decreases in output and income were the most significant among subsistence farmers (followed by commercial farming) in the quantitative data (i) of Morogoro; with only qualitative data (ii) showing the strongest effect on commercial farms.

### Information technology usage since the outbreak of COVID-19

Access to agricultural information on inputs, agronomic advice, and market information has not changed since the outbreak of COVID-19. Interestingly, the quantitative data (i) reveals a gap between the perception of IT use by farmers and experts. While agricultural experts saw a positive development in the use of IT, smallholders did not report any change. The qualitative data (ii) proves this as 16 out of 20 respondents disagreed with the statement that there was an increase in IT usage to obtain information for market searches of agricultural produce since the outbreak of COVID-19.

Only 20 % of respondents in Mafinga confirmed that they started using digital technology to obtain agronomic and market information, namely, WhatsApp and emails. Among them, two respondents are graduates and one added that he received advice from his former university teacher. The larger share of respondents, who did not see a change in access and use of information technology, argued that all agricultural extension officers were positioned in urban areas and did not visit rural areas. Several smallholders argued that most rural farmers do not have access to smartphones and, as important information is mainly spread via programs on the internet, most smallholder farmers cannot be reached.

### Barriers experienced in adapting to the new situation

All respondents confirmed that government rules regarding social interactions had changed since the outbreak of the pandemic, specifically those regarding social distancing, wearing masks, and washing hands/sanitizing. For cultural reasons and a lack of awareness in the communities, the respondents questioned the effectiveness of these measures. On the one hand, they explained that, in most cases, people were not following all measures but mixing them arbitrarily, such that someone is wearing a mask but not keeping a distance or they are sanitizing but not wearing a mask.

Some respondents complained that they could not purchase masks and that the government was not providing or subsidizing them, which is why they were relying on those masks given out by NGOs working in the localities. Other respondents associated the increased living costs with using too much water for washing hands. Small traders and some household members argued that it was expensive to keep enough water available to ensure that people wash their hands before they enter their shops or houses.

On the other hand, respondents pointed out that people were still gathering for cultural events and could not maintain social distance due to the nature of society and prevailing ignorance. About 90% of the respondents confirmed that culture was the main barrier in adapting to new changes. The majority of respondents (87%) commented that they recommend following the three major strategies (masking, distancing, and sanitization) by the government as they were believed to be scientifically sound, ensuring greater safety. However, with regard to the lack of awareness, 15% of respondents also recommended that the government should improve public education campaigns that explain how to prevent the pandemic. Overall, about 60% of the respondents complained that the government was neglecting them. 

The poor COVID-19 governance in Tanzania, especially during the first phase under the government of John Magufuli, put citizens at risk as the principal proposed measure against the virus was prayer, with the majority of citizens denying the existence of the pandemic. As of December 2021, it was still not clear how many people died or were affected by and survived the pandemic, as reports of cases are still sporadic. This was a stumbling block for future planning.

###  Resilience building for smallholder farmers

While Nchanji et al. (2021) [17] propose the provision of subsidies for agricultural inputs as their first policy recommendation, this study suggests that the government could help Tanzanian smallholder farmers build resilience by providing easier access to, and the provision of, long term loans. With a view of the coping mechanisms of smallholders during the pandemic, findings confirm that most strategies include financial coping, e.g. borrowing money from family members. Furthermore, it is known that institutional and community support plays an important role in reducing dependencies on external actors during emergencies. Communities in Tanzania often share food products and labor, while also receiving remittances from family members living abroad. Combined, these all facilitate enhanced coping. In this case, it was a group of farmers in the study areas who were more dependent on markets and were significantly affected by logistical bottlenecks in the value chain, reduced market demands, and reduced purchasing power. Remittances were affected because family members living abroad were also affected by the pandemic. Furthermore, since the outbreak of COVID-19, Smallholder farmers in Mafinga also requested subsidies for agricultural inputs like fertilizers and water in order to challenge the financial limitations they were facing and to improve their livelihood resilience during future pandemics/shocks Some respondents added that the government should give help to smallholder farmers to allow them to search for a market for their products, as the problems mentioned in the Mafinga region show dependency on middle persons, poor storage of perishable fruits, and impeded market access. It is advised that the Tanzanian government work with stakeholders, including researchers, community organizations, and funding organizations. This is important to identify weaknesses, choke points, and vulnerabilities in agriculture and food systems. It will also help to identify critical services that need to be strengthened in order to increase preparedness for systemic risks and identify opportunities to strengthen networks between public and private stakeholders. Rural capacity building and affordable infrastructure are important for farmers to adapt to challenges with the help of new technologies. Government should strive to support accessible and affordable infrastructure, such as the use of ITs for early warning systems and as a way for market searching during crises. Centralization shows that when there is a lockdown, distribution, movement, and transportation gets out of order. Therefore, finding a way around market closures and ensuring delivery is one way to keep the food supplies moving and farmers in business.

## Conclusion & Recommendations

The study explores the impacts of COVID-19 on smallholder farmers in two regions of Tanzania. The research finds decreased agricultural production and income caused by the outbreak of COVID-19 in the selected communities in Tanzania. Both regions are places where agriculture is typified as cash crop/commercial agriculture and where many interactions between local smallholder farmers and external traders take place. Thus, at the international level, lockdown measures implemented by other countries affected smallholder farmers’ market opportunities. COVID-19 reduced market opportunities for smallholder farmers in both study areas because of restricted international mobility leading to decreased income. These communities' vulnerabilities became increasingly complicated due to income reductions resulting from the outbreak of COVID-19. This suggests the need for a working partnership between stakeholders that educate and build the capacity of smallholders to diversify economically, in order to cope with uncertainties.

Small traders experienced difficulty transporting products to local and urban food markets, with reduced employment in agriculture because of reduced economic activity resulting from border closures, voluntary isolation due to fear of the spread of COVID-19, and the general decreased purchasing power of Tanzanians. Food security was impacted in that food availability was reduced due to mobility restrictions and reduced incomes. Despite providing better access to information for smallholder farmers, information technology did not work as a coping strategy, because its access, e.g., through smartphones, is restricted and, thus, adoption in rural Tanzania is limited.

Although Tanzania did not impose a total lockdown, the country was affected by COVID-19, partly by policies of other countries. Impacts included: (i) a decline in local markets as smallholder farmers had fewer trading partners from neighboring states; (ii) a loss of employment opportunities due to the absence of both local and external trade; (iii) reductions of farm output and income; (iv) a lack of agricultural inputs (fertilizer etc.) that are usually imported; (v) fear to continue farming activities due to news about COVID-19 spreading; and (vi) reduction of work efficiency because of fewer social gathering due to voluntary isolation. To analyze the impact of COVID-19 on all components of the food system in Tanzania, further research is necessary. As impacts on the environment and processing go beyond the scope of this study, focusing on the latter would bring important information gains.

Based on the findings, this research recommends the following**:**

- To help smallholder farmers in financial crisis, easier access to, and provision of, small long-term loans are essential.
- To offset the impacts of high prices of agricultural inputs, governments in collaboration with other countries and multilateral companies should secure and build on positive trade facilitation steps to reduce distortions at the border, to reinforce the role that global markets can play in ensuring stable food supplies.
- To ensure an effective flow of information at all levels, the government should invest in data systems at the local, national, and global levels so that real-time information can be made available to decision-makers, helping them to increase confidence in supply during crises. For this, access to information technology in rural areas is indispensable and should be subsidized by the government.
- To ensure an effective flow of food products, international collaboration should foster tiered mobility restrictions allowing travel for economic reasons during crises. Trade barriers against food products most affected by the pandemic should be lowered [20]. Working on common guidelines and the harmonization of containment and distancing rules with neighboring countries is recommended [27]. To support farmers and their organizations, it is important to allow the movement of seasonal workers and transport operators. For this, identifying collection centers closer to producers and developing storage facilities such as warehouse receipt system platforms are keys for farmers so that they can deliver their products without the need to go to markets.

Overall, policymaking and market management will have to balance the protection of the health of citizens with the substantial economic and political threats to the economy resulting from the COVID-19 policy response. This balancing act can be considered an ongoing process in which adjustments to health and protection policies, on the one hand, and economic and support policies on the other, have to be made continuously, as suggested by Wieck et al. [27]. Identifying threats, such as border closures, the occurrence of domestic consumption dominance, and the question about regionalism of production in the debate, as areas needing regular review and adjustment. Above all, however, the health and food security of future generations, therefore, the averting of causes of climate change, must be considered just as much when making policies during crises.

## Data Availability

All lists of materials used data recorded, generated and analyzed during this study are included in this study. Separate documents are available upon request from the corresponding Author.

## Abbreviations

BOT: Bank of Tanzania
COVAX: COVID-19 Vaccines Global Access E
CLAC: Economic Commission for Latin America and the Caribbean
EU: European Union
FAO: Food and Agriculture Organization
NGOs: Non-Government Organizations
IT: Information technology
SADC: Southern Africa Development Coordination
TVs: Televisions
UN: United Nations
UNICEF: United Nations Children’s Fund
UNDP: United Nations Development Programme
USA: United States of America
WHO: Word Health Organization
COVID-19: Corona virus disease
